# Psychometric characterization of the obstetric communication assessment tool for medical education: a pilot study

**DOI:** 10.5116/ijme.5740.4262

**Published:** 2016-06-11

**Authors:** A. Noel Rodriguez, Peter DeWitt, Jennifer Fisher, Kirsten Broadfoot, K. Joseph Hurt

**Affiliations:** 1 Department of Obstetrics and Gynecology, Divisions of Maternal Fetal Medicine and Reproductive Sciences, University of Colorado School of Medicine, Anschutz Medical Campus, Aurora, Colorado, USA; 2 Biostatistics and Informatics, Research Consulting Lab, Colorado School of Public Health, Aurora, Colorado, USA; 3 Center for Advancing Professional Excellence (CAPE), University of Colorado School of Medicine, Aurora, Colorado, USA

**Keywords:** Patient-centered communication, undergraduate medical education, standardized patient, inter-rater reliability, psychometrics

## Abstract

**Objectives:**

To characterize the psychometric properties of a novel Obstetric Communication Assessment Tool (OCAT) in a pilot study of standardized difficult OB communication scenarios appropriate for undergraduate medical evaluation.

**Methods:**

We developed and piloted four challenging OB Standardized Patient (SP) scenarios in a sample of twenty-one third year OB/GYN clerkship students: Religious Beliefs (RB), Angry Father (AF), Maternal Smoking (MS), and Intimate Partner Violence (IPV). Five trained Standardized Patient Reviewers (SPRs) independently scored twenty-four randomized video-recorded encounters using the OCAT. Cronbach’s alpha and Intraclass Correlation Coefficient-2 (ICC-2) were used to estimate internal consistency (IC) and inter-rater reliability (IRR), respectively. Systematic variation in reviewer scoring was assessed using the Stuart-Maxwell test.

**Results:**

IC was acceptable to excellent with Cronbach’s alpha values (and 95% Confidence Intervals [CI]): RB 0.91 (0.86, 0.95), AF 0.76 (0.62, 0.87), MS 0.91 (0.86, 0.95), and IPV 0.94 (0.91, 0.97). IRR was unacceptable to poor with ICC-2 values: RB 0.46 (0.40, 0.53), AF 0.48 (0.41, 0.54), MS 0.52 (0.45, 0.58), and IPV 0.67 (0.61, 0.72). Stuart-Maxwell analysis indicated systematic differences in reviewer stringency.

**Conclusions:**

Our initial characterization of the OCAT demonstrates important issues in communications assessment. We identify scoring inconsistencies due to differences in SPR rigor that require enhanced training to improve assessment reliability. We outline a rational process for initial communication tool validation that may be useful in undergraduate curriculum development, and acknowledge that rigorous validation of OCAT training and implementation is needed to create a valuable OB communication assessment tool.

## Introduction

Effective patient-centered communication correlates positively with patient satisfaction and adherence to medical treatment, independent of treatment outcomes.^1^^-^^3^ The Association of American Medical Colleges (AAMC) suggests “a planned and coherent framework for communication skills teaching” with assessment of students’ communication abilities and efficacy of educational programs. The Kalamazoo Consensus Statement on medical education lists essential tasks for communication training, including building the doctor-patient relationship, opening the discussion, gathering information, understanding the patient perspective, sharing information, reaching agreement on problems and plans, and providing closure.^4^

Structured patient-physician communication training is inconsistently integrated in undergraduate medical education curricula. The AAMC reports wide variation in educational methods, with primary approaches to communication training including small group discussion, lectures, and interview of standardized and real patients in simulated encounters.  Effective, validated teaching materials and evaluation instruments for this topic are needed. The Calgary-Cambridge Observation Guides is a common framework for medical communication teaching and assessment.^5^ The Guides emphasize the physician-patient relationship and serve as a comprehensive tool to teach medical interviewing but are not designed or validated for critical discrimination or testing.^6^

Objective Structured Clinical Examinations (OSCE) using standardized patient actors provide low-risk opportunities to evaluate communication skills and develop clinical competence.^7^ Evaluation of student performance typically uses a structured assessment measure. Ideally the instrument is consistent with educational goals and objectives, easy to implement, and demonstrates psychometric rigor including internal consistency (IC), inter-rater reliability (IRR), and construct validity.^8^ Existing communication measures vary in content and form,^9^^-^^16^ and many are not well tested for reliability or validity.^17^ Without validated assessment tools, it is difficult to determine the efficacy of existing communication training programs or novel educational interventions.

Difficult communication occurs in all medical specialties. Despite this, practitioners report inadequate formal training in discussing the most difficult topics or delivering bad news.^18^^, ^^19^ Educational models using SP role-playing are shown to improve students’ comfort with imparting difficult news such as a new cancer diagnosis.^20^ OB clinical encounters frequently address challenging topics that require careful communication and enhanced sensitivity,^21^^, ^^22^ but focused OB communication training is limited in undergraduate medical education and relevant validated assessment tools are lacking.^23^^,^^24^ Further, the unique situation in obstetrics with both adult and fetal patients warrants investigation and development of a valid obstetric communication instrument.

We describe the development of OB-focused challenging communication cases and our initial characterization of the Obstetric Communication Assessment Tool (OCAT). Our instrument is derived from the Calgary-Cambridge Observation Guides,^6^^,^^25^ and will ultimately be used to evaluate a novel community-based OB communication curriculum we have developed.  Here, we report case preparation, trial and revision, initial SP training, and the use of video-recorded encounters to assess OCAT psychometrics. We discuss IC and IRR and suggest training and analysis approaches to further improve our psychometric measures. We present a structured process for SP case development and for quantitative validation of a communication assessment tool that may be employed across medical specialties and by other communications curriculum developers.

## Methods

### Case scenario preparation

**Figure 1:**
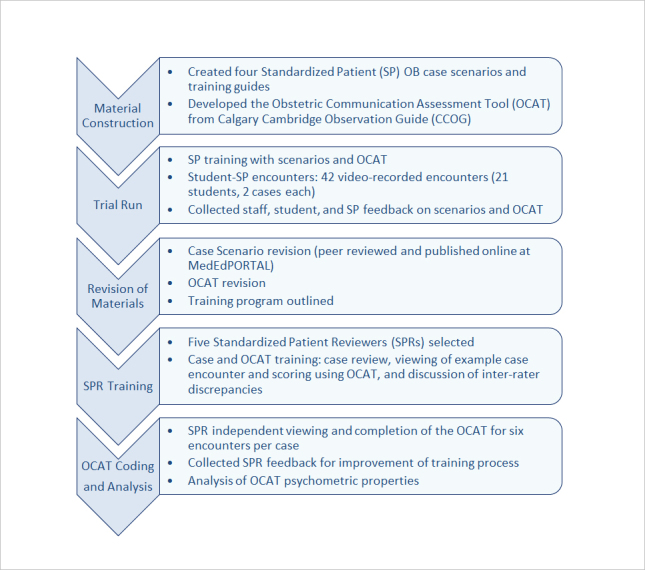
Communication module development schematic

Four SP cases were written to reflect difficult OB-based encounters: Religious Beliefs (RB), Angry Father (AF), Maternal Smoking (MS), and Intimate Partner Violence (IPV Appendix 1).^26^ The cases are high in emotional content requiring exploration of underlying patient concerns and higher order communication skills, and were developed from needs-assessment interviews with local families participating in a March of Dimes family fair.  We chose cases that were common among families having experienced a high risk pregnancy, and reviewed with maternal fetal medicine faculty who confirmed these are not extremely rare cases.  Case scenarios were reviewed and edited by co-authors with expertise in communication pedagogy and SP testing, and by a practicing obstetrician for detail and accuracy. Each scenario was prepared using detailed SP templates from our Center for Advancing Professional Excellence (CAPE) facility at the University of Colorado Anschutz Medical Campus. Cases were revised using student, staff, and SP feedback from the initial trial run (see Trial Run, below) and have been published separately for secure access by medical educators.^26^  Figure 1 summarizes case and OCAT development.  This manuscript primarily reports results from the last two parts of OCAT development: SP training and OCAT evaluation and psychometrics.

### Obstetric Communication Assessment Tool (OCAT) Development

The OCAT consists of common and unique communication elements for each case. Twenty-six common communication items were selected from the Calgary Cambridge Observation Guides under six broad categories: Initiating the Session, Gathering Information, Building the Relationship, Providing Structure, Sharing Information: Explanation and Planning, and Closing the Session (Appendix 2). Five to ten additional unique items were created to assess goals specific to each case (Appendix 3). These case-specific items orient SPs to key elements of the case and document required skills that indicate a successful encounter. For example, asking about patient safety in a context highly suspicious for intimate partner violence is necessary to identify the underlying and most important issue of that case. Each checklist concludes with two items on overall performance that summarize: 1) the student’s ability to convey patient advocacy; and 2) the SP’s willingness to interact with the student as a patient again (Appendix 2). Overall, our instrument purports to measure comprehensive patient-physician communication, with additional items for obstetric concerns. Our goal was to keep the checklist as brief as possible while maintaining discriminatory capacity and enough detail to provide useful feedback for learners, if desired.

SPs respond to scaled items by classifying a particular skill or ability as “NOT demonstrated during this encounter”, “PARTLY, but inconsistently/incompletely demonstrated”, “MOSTLY, but inconsistently/incompletely demonstrated”, or “COMPLETELY/consistently demonstrated”.  SPs were trained to score first according to an “all or none” principle, meaning they would first consider whether the skill was “not demonstrated” (0%) or “completely demonstrated” (100%). If neither applied, they would discern “partially demonstrated” (≤50%) from “mostly demonstrated” (>50%) on their assessment of skill performance. Our four-category scale is intended to enhance discriminatory capacity compared with a three-category instrument used previously at our institution, without extensive additional training. Certain items required a “Yes” or “No” response and one item included a “Not Applicable” response (Appendix 2 and 3).

After a trial run of student-SP encounters (see Trial Run, below), a study author reviewed all student recordings for each case scenario and independently scored student performance using the OCAT. Items that were discrepant between the researcher and the SPs were evaluated for ambiguous language and edited for clarity. Free text comments from both learners and SPs were considered and redundant items eliminated. Feedback from SPs, CAPE faculty, and students was incorporated and a revised OCAT prepared. This final OCAT was used in our subsequent investigation below (see OCAT Characterization and Validation, below).

### Trial run

SPs employed and trained by the CAPE were selected for their ability to portray case scenarios, interact with students during a simulated clinical encounter, and assess student performance. Participating SPs had over two years experience working in a standardized testing environment and signed a non-disclosure confidentiality agreement upon hire. They attended group training sessions for each of our four cases led by experienced CAPE trainers and the study researchers. Training included reading and detailed review of the case, role-playing practice, discussion of assessment objectives, and instruction in appropriate use of the OCAT.

Twenty-one third year medical students in the Women’s Care (OB/GYN) clerkship at the University of Colorado School of Medicine participated in our OSCE pilot held at the CAPE. Students had completed half of the required clinical clerkships and had a variety of previous clinical exposures. We assigned students to participate in two of four SP encounters at the conclusion of their clerkship. In total, twelve students completed the IPV and MS cases and nine students completed the AF and RB cases. Each encounter consisted of a five-minute preparatory period when students reviewed a brief scenario before entering the room, fifteen minutes for the SP encounter, and ten minutes to complete a computerized self-evaluation questionnaire outside of the room. In the ten-minute intermission after each encounter, the SP completed an OCAT online using the EMS SimulationiQ™ web-based data capture system in the encounter room. They were instructed to complete the assessment form in its entirety prior to the beginning of the next simulated encounter. Each encounter was video-recorded and stored for future review and training using EMS SimulationiQ™. 

### OCAT Characterization and validation

A study author independently reviewed all video-recordings and identified high, medium, and low student performers for each case. Two examples of each level were selected for review and scoring by SP Reviewers (SPRs). Estimates of OCAT mean and variance for each case were determined. We calculated that five SPRs would need to review six encounters for each case, yielding 30 evaluations per case, to achieve 99% power to reject our null hypothesis versus the alternative hypothesis of Cronbach’s alpha ≥0.7, with alpha error at 5%.  Cronbach’s alpha of 0.7 was selected as our first psychometric outcome of interest, using the lower end of the 0.7-0.8 threshold for acceptable IC.^27^

We recruited five CAPE-employed SPs, different from those who participated in the initial trial run, to independently review and score the selected video-recorded encounters.  These were experienced male or female SPs from our testing center; all had more than 2 years of standardized patient and medical student evaluation experience.   All were college educated or currently enrolled in college level classes. Standardized Patient Reviewers (SPRs) participated in a three-hour training session for the first case and two-hour sessions for each subsequent case, conducted by experienced CAPE staff and the study investigators. Each session consisted of a thorough review of the SP case scenario, detailed review of the revised OCAT, discussion of the scoring rubric, and clarification of expected skills and abilities associated with each item. SPR trainees practiced scoring one student-SP video encounter during each session. They discussed their scores with the trainers and each other, emphasizing discordant items, with the goal of aligning SPR scores with expected responses.

One week after the training session for each case, the SPRs independently viewed six randomized student-SP video-recordings for the corresponding case and immediately completed an OCAT for each encounter. The measure was again completed in the EMS SimulationiQ™ platform. SPRs were instructed to view the encounter only once, to refrain from rewinding the recording, and to complete the measure only after viewing the entire encounter, intending to mimic the live SP testing environment.

### Statistical methods

Scaled items were coded on a scale of zero to three. Zero points were awarded for “NOT demonstrated”, one point for “PARTLY demonstrated”, two points for “MOSTLY demonstrated”, and three points for “COMPLETELY demonstrated.” The “Yes/No” items were coded numerically as three points for “Yes” and zero points for “No”, with the exception of one item on the MS case (Appendix 3): Maternal Smoking, Item 4), which was reverse coded due to negative voicing of the item. Question number 16 (Appendix 2) was not used for data analysis due to the option of “Not Applicable,” which is a non-ordinal value incompatible with computation of Cronbach’s alpha and Intraclass Correlation Coefficient-2 (ICC-2).

Assessment of the OCAT’s internal consistency (IC) was performed via Cronbach’s alpha, with confidence intervals calculated using the method of Feldt et al; high values indicate measurement of a single construct by the SPR within each case.^28^^,^^29^ SPR inter-rater reliability  was determined by ICC-2, a measure of agreement between random samples of averaged ratings.^30^^,^^31^ Further investigation of agreement between SPRs was conducted using the Stuart-Maxwell test.^32^^,^^33^ This test is a generalization of the McNemar test, which is used to determine if discordant pairs of observations tend to be over or under-rated.  This test is useful to determine whether systematic but internally consistent differences in scoring among individual SPRs are the cause of the observed excessive interrater variation.

Analysis was completed in R version 3.1.0 (2014-04-10). Cronbach’s alpha was calculated with the “psych” package, while ICC and the Stuart-Maxwell tests were performed in the “irr” package.^34^^-^^36^ The results are reported along with 95% confidence intervals. 

### Ethics and privacy

The Colorado Multiple Institutional Review Board (COMIRB) approved this project. All students sign a standard CAPE consent giving permission to record and store all student-SP encounters for education and research purposes. All data and assessment materials were de-identified and stored in a secure location using a unique identification number. SPs and SPRs sign a confidentiality agreement prior to the study.

## Results

### OCAT Internal Consistency (IC)

Cronbach’s alpha estimates and 95% confidence intervals are reported in Table 1. IC for each case and subscale analyses are reported. Overall, the RB, MS, and IPV cases demonstrated excellent Cronbach’s alpha values, indicating high IC within each SPR and across the measure. For the RB case, question number two (Appendix 2 – Initiating the Session) and number six (Appendix 3 – RB) had no variance and were therefore omitted from the analysis; the estimated Cronbach’s alpha was 0.91.  Similarly, in the AF case, question number 27 (Appendix 2 – Overall Student Performance) and number two (Appendix 3 – AF) lacked variance and were omitted, with an acceptable Cronbach’s alpha of 0.76. For the MS and IPV cases, no items were omitted and alphas were estimated at 0.91 and 0.94, respectively. Nine of the 27 subscale analyses demonstrated good to excellent alpha values. Seven met acceptable criteria, and eleven showed poor IC likely due to the limited number of items within those subscales.

**Table 1:** Estimated Cronbach alpha values for each case overall and each subscale

Case	Religious Beliefs (RB)		Angry Father (AF)		Maternal Smoking (MS)		Intimate Partner Violence (IPV)	
	N	Alpha (CI)	N	Alpha (CI)	N	Alpha (CI)	N	Alpha (CI)
Whole instrument	36	**0.91**(0.86, 0.95)	33	**0.76**(0.62, 0.87)	36	**0.91**(0.86, 0.95)	38	**0.94**(0.91, 0.97)
Subscales								
Initiating the Session	4	**0.60** (0.33, 0.78)	5	0.44 (0.08, 0.70)	4	0.38 (-0.03, 0.67)	4	0.43 (0.05, 0.69)
Gathering Information	8	**0.80** (0.68, 0.89)	7	0.49 (0.17, 0.72)	8	**0.77** (0.62, 0.87)	9	**0.86** (0.78, 0.93)
Building the Relationship	6	**0.89** (0.81, 0.94)	9	0.52 (0.22, 0.74)	6	**0.93** (0.88, 0.96)	6	**0.87** (0.79, 0.93)
Providing Structure	2	-0.57 (-1.67, 0.17)	3	-0.22 (-1.05, 0.35)	2	0.53 (0.20, 0.75)	2	0.26 (-0.26, 0.61)
Sharing Information	12	**0.60** (0.36, 0.78)	5	0.44 (0.08, 0.70)	12	**0.65** (0.44, 0.81)	13	**0.87** (0.79, 0.93)
Closing the Session	2	**0.73** (0.55, 0.86)	2	0.52 (0.18, 0.74)	2	**0.65** (0.41, 0.82)	2	**0.82** (0.69, 0.90)
Overall Performance	2	**0.81** (0.68, 0.90)	2	Not estimable	2	**0.62** (0.35, 0.80)	2	**0.82** (0.69, 0.90)

Note: N is the number of items for each particular instrument or subscale. Cronbach’s alpha estimates and 95% confidence intervals are listed. Values of internal consistency (IC) can be interpreted as follows: alpha 0.90 indicates excellent IC. Bold values are those in the acceptable range or above.

**Table 2:** Estimated Intraclass Correlation Coefficient-2 (ICC-2) for each case overall and each subscale

Case	Religious Beliefs (RB)		Angry Father (AF)		Maternal Smoking (MS)		Intimate Partner Violence (IPV)	
	N	ICC (CI)	N	ICC (CI)	N	ICC (CI)	N	ICC (CI)
Whole instrument	36	0.46 (0.40, 0.53)	33	0.48 (0.41, 0.54)	36	0.52 (0.45, 0.58)	38	**0.67** (0.61, 0.72)
Subscales								
Initiating the Session	4	0.19 (0.04, 0.41)	5	0.39 (0.23, 0.58)	4	0.59 (0.41, 0.76)	4	0.28 (0.11, 0.50)
Gathering Information	8	**0.64** (0.52, 0.75)	7	0.36 (0.22, 0.52)	8	0.52 (0.39, 0.65)	9	**0.64** (0.53, 0.75)
Building the Relationship	6	0.30 (0.14, 0.50)	9	0.26 (0.13, 0.41)	6	0.53 (0.37, 0.70)	6	0.53 (0.37, 0.70)
Providing Structure	2	0.42 (0.17, 0.72)	3	0.21 (0.03, 0.47)	2	0.59 (0.34, 0.83)	2	0.54 (0.29, 0.80)
Sharing Information	12	0.40 (0.29, 0.52)	5	0.54 (0.37, 0.70)	12	0.47 (0.36, 0.58)	13	**0.66** (0.57, 0.75)
Closing the Session	2	-0.12 (-0.19, -0.07)	2	0.02 (-0.12, 0.31)	2	0.35 (0.10, 0.67)	2	0.11 (-0.06, 0.44)
Overall Performance	2	0.32 (0.08, 0.64)	2	0.40 (0.15, 0.71)	2	0.22 (0.01, 0.56)	2	0.49 (0.24, 0.77)
Case	number of items analysed n	ICC (CI)

Note: N is the number of items for each particular instrument or subscale. ICC-2 estimates and 95% confidence intervals are listed. Values of ICC-2 can be interpreted as follows: ICC

### OCAT Inter-Rater Reliability (IRR)

ICC-2 values with 95% confidence intervals are reported in Tables 2-4. Overall, the reliability among SPRs is low on all subscales (Table 2). For the RB, AF, and MS cases, the estimated ICC-2 values suggest that reliability among SPRs is unacceptable, with values of 0.46, 0.48, and 0.52, respectively. The ICC-2 value for the IPV case is estimated at 0.67, demonstrating poor reliability (Table 2).  Additional ICC-2 values were calculated separately for the common items only (Table 3), but reliability remained unacceptable, without improvement compared to the whole instrument, suggesting that even the common Calgary-Cambridge based elements did not attain sufficient reliability among our SPRs.

We hypothesized that unacceptable ICC-2 scores may be due to SPRs being unable to discriminate incompletely demonstrated skills. Therefore, we re-analyzed the responses after combining responses of “PARTLY demonstrated” and “MOSTLY demonstrated” (Table 4). This simplification did not improve IRR, likely indicating inconsistent application of scoring criteria or systematic differences in SPR scoring approach.  The study sample size was calculated based upon Cronbach’s alpha, not ICC-2, so our small sample size may partly explain the poor IRR performance.

**Table 3:** Estimated Intraclass Correlation Coefficients-2 (ICC-2) for common items

Case	number of items analysed n	ICC (CI)
Whole Instrument	28	0.45 (0.41, 0.49)
Subscales		
Initiating the Session	4	0.50 (0.41, 0.60)
Gathering Information	7	0.40 (0.32, 0.49)
Building the Relationship	6	0.39 (0.31, 0.48)
Providing Structure	2	0.42 (0.29, 0.57)
Sharing Information	5	0.52 (0.44, 0.61)
Closing the Session	2	0.12 (-0.02, 0.26)
Overall Student Performance	2	0.31 (0.19, 0.46)

Note: Common items are similar across all four cases, including the core 26 scaled items and two Overall Student Performance questions. ICC-2 estimates and 95% confidence intervals are listed. Values of ICC-2 can be interpreted as follows: ICC

**Table 4:** Estimated Intraclass Correlation Coefficient-2 (ICC-2) using consolidated scale for each case overall and for each subscale

Case	Religious Beliefs (RB)		Angry Father (AF)		Maternal Smoking (MS)		Intimate Partner Violence (IPV)	
	N	ICC (CI)	N	ICC (CI)	N	ICC (CI)	N	ICC (CI)
Whole Instrument	36	0.40 (0.33, 0.47)	33	0.42 (0.35, 0.49)	36	0.46 (0.39, 0.52)	38	0.63 (0.58, 0.69)
Subscales								
Initiating the Session	4	0.11 (-0.02, 0.31)	5	0.44 (0.28, 0.63)	4	0.53 (0.35, 0.72)	4	0.22 (0.06, 0.45)
Gathering Information	8	0.43 (0.30, 0.58)	7	0.17 (0.06, 0.33)	8	0.33 (0.20, 0.48)	9	0.58 (0.46, 0.70)
Building the Relationship	6	0.29 (0.13, 0.49)	9	0.29 (0.16, 0.44)	6	0.43 (0.26, 0.61)	6	0.44 (0.27, 0.62)
Providing Structure	2	0.58 (0.32, 0.82)	3	0.10 (-0.04, 0.35)	2	0.50 (0.24, 0.78)	2	0.62 (0.37, 0.84)
Sharing Information	12	0.38 (0.28, 0.50)	5	0.46 (0.29, 0.64)	12	0.45 (0.34, 0.56)	13	0.65 (0.56, 0.74)
Closing the Session	2	-0.07 (-0.17, 0.16)	2	0.06 (-0.10, 0.37)	2	0.40 (0.15, 0.71)	2	0.06 (-0.09, 0.38)
Overall Performance	2	0.27 (0.05, 0.61)	2	0.40 (0.15, 0.71)	2	0.19 (-0.01, 0.53)	2	0.40 (0.15, 0.71)

Note: The consolidated scale combines middle responses of “Partly, but incompletely/inconsistently demonstrated” and “Mostly, but incompletely/inconsistently demonstrated”. N is the number of items for that particular instrument or subscale. ICC-2 estimates and 95% confidence intervals are listed. Values of ICC can be interpreted as follows: ICC

### Evaluator comparison

To further explore the basis for OCAT’s excellent IC but low IRR, we performed a Stuart-Maxwell analysis of paired evaluator discordance (Table 5). We found systematic differences in scoring by individual SPRs. SPR 3 and 4 overall provided equivalent scores and higher than the other evaluators. SPR 1 gave higher scores than SPR 2, who gave higher scores than SPR 5. This indicates that the poor IRR may be attributed to persistent differences in SPR rigor and inconsistent operationalizing of the scoring system.

**Table 5:** SPR rigor as measured by the Stuart-Maxwell test

SPR X vs. SPR Y	X<Y	Same Rating	X>Y	p-value
SPR 1 vs. SPR 2	13.5%	62.2%	24.2%	<0.0001
SPR 1 vs. SPR 3	27.6%	57.6%	14.9%	<0.0001
SPR 1 vs. SPR 4	31.5%	54.8%	13.7%	<0.0001
SPR 1 vs. SPR 5	13.4%	60.8%	25.8%	<0.0001
SPR 2 vs. SPR 3	32.7%	57.4%	9.8%	<0.0001
SPR 2 vs. SPR 4	36.0%	54.7%	9.4%	<0.0001
SPR 2 vs. SPR 5	18.1%	62.1%	19.8%	<0.0001
SPR 3 vs. SPR 4	24.1%	56.8%	19.1%	0.1246
SPR 3 vs. SPR 5	10.6%	52.5%	36.9%	<0.0001
SPR 4 vs. SPR 5	9.4%	51.2%	39.4%	<0.0001

SPR X versus Y: Percentage of responses where the two noted SPRs provided the same response (Same Rating), SPR X provided a lower rating than SPR Y (X<Y) , and SPR X provided a higher rating than SPR Y (X>Y) for all assessment items. From highest to lowest scorers: SPR 3 = SPR 4 > SPR 1 > SPR 2 >SPR 5.

## Discussion

Validated and well-characterized tools to assess provider communication are required to create evidence-based curricula for patient-centered care. Here, we have described the characterization of the OCAT for evaluation of novel standardized OB communication scenarios. Dedicated OB communication modules are lacking in undergraduate medical education, despite the uniquely sensitive and complex situations to be explored. Objective assessment measures are essential to ensure efficacy and utility of training programs. To address that, we performed initial psychometric characterization of the OCAT.

The OCAT demonstrates excellent IC, as measured by Cronbach’s alpha, for three of four cases and acceptable IC for the AF case. Overall, this indicates high construct consistency. We speculate that the lower IC for the AF case may be attributed to the complexity of two SP actors in that scenario (the patient and her partner). However, as Cronbach’s alpha is sensitive to the number of items used for analysis, fewer case-specific items in the AF case may influence our results.  In our analysis, ≥36 assessment items appears to be optimal.

The OCAT yields unacceptable IRR for all cases, as measured by ICC-2, though there is an important distinction between rater consistency and agreement. Consistency describes reliability in relative score rankings, although the scores may not be identical. The ICC-2 assesses agreement, for which raters must independently assign identical scores.^37^ Poor IRR could be due to systematic differences between raters in assigning scores, inadequate training, inability of the scale to characterize the full continuum of communication skill, or the inherent difficulty in objectively quantifying the subjective interpretation of patient-centered communication. Paired-rater analysis, however, indicates a systematic difference in the stringency of our SPRs. If this is true, reliability may be improved by changes in our training protocol.

OCAT IRR was not improved by simplification of the scale or by separate analysis of common OCAT items (Appendix 2). We hypothesized that consolidating the middle responses on our scale (“PARTLY demonstrated” and “MOSTLY demonstrated”) would improve reliability, as this was the primary source of inter-rater discordance during training. Surprisingly, IRR worsened, indicating that rater discordance was distributed across the scale and strongly suggests the need for more extensive and rigorous SP training. Further, we anticipated that common items, which reflect generalized communication skills familiar to our SPRs and similar to other assessment instruments would more reliably score student performance. Our analysis did not support this, and given that ICC-2 is sensitive to the number of items, may have been limited by the exclusion of case-specific questions. Neither case-specific or general questions alone showed acceptable interrater reliability.  We suspect that the lack of consistency among SPRs may be a common problem across institutions with varied local SP training practices.

Rigorous training has been shown to improve IRR. SP training for high-stakes clinical examinations, such as for the National Board of Medical Examiners (NBME), consists of several full days of intensive training, including verification of accuracy and consistency before graduating to real student-SP testing encounters.^38^^,^^39^  Jensen, et al, described a training process involving 18 hours of instruction followed by independent coding until raters attained an IRR of at least 0.7. Raters met on a weekly basis to discuss their evaluations and IRR was measured at regular intervals to ensure persistent reliability. Analysis of IRR over the first twenty video-recorded encounters compared to the next thirty encounters demonstrated improvement with practice.^13^ Another successful approach, by Krupat, et al, used an extensive training guide with example behaviors and corresponding scores to decrease rating subjectivity. Approximately eight to ten hours of training was needed to achieve reliability in that study.^40^ Our training method, with a maximum of three hours training per case, and without reliability verification, appears to be inadequate, though this training regimen may be typical for many institutions. We are now developing a video-based, interactive training module for the OCAT, and will test whether that approach improves our instrument reliability.   It is possible, as well, that our emotionally difficult cases may be more difficult for SPs to perform well and then score accurately.  We are conducting further studies to determine whether this is the case, but feedback from our SPRs did not suggest this was a factor.

Strengths of our study include the use of a well-established model of medical communication (CCOG), collaboration with medical communication education and clinical experts for OCAT development, and inclusion of student, faculty, and staff feedback for case and tool revision. Our design utilized a wide range of student abilities and randomized video presentation to the SPRs.  Our statistical analyses help explain the low IRR. Although there may be differences between video and live encounter SP experiences, our approach using trained SP raters to assess video-recorded encounters is well-described and validated.^12^^-^^14^^, ^^40^

A limitation of our study is the small number of student encounters reviewed, although the number of SPRs and videos was supported by a priori sample size considerations. Future study of the OCAT may benefit from increasing the number of reviewers and video-recorded cases. Further, we did not assess accuracy (e.g. comparing SPR ratings to a gold standard correct response). Although accuracy is not typically reported for new instruments, it can assist developers in identifying areas of discrepancy between novice and expert raters during training.^37^ We are developing expert coded sets of student-SP video recordings, similar to the process described by Lang et al, for expert-novice rater comparison and SP training.^14^

Published OB SP modules focus on procedural and technical skills, and either address communication as a secondary issue or, more commonly, do not specifically assess communication at all.^41^^-^^43^ Colletti et al reported improved clinical performance after students completed challenging SP clinical scenarios with patients experiencing spontaneous pregnancy loss or a new diagnosis of rectal cancer, but the method to ascertain clinical performance was not clear.^24^ Additionally, the generalizability of routine medical communication training across specialties is not extensively studied, and it is unknown whether general medical communication training is transferable to OB encounters, particularly in challenging scenarios.^44^ Further, if we are able to train our SPs to acceptable and discriminatory reliability, we plan to examine the correlation of our OCAT assessment with standard student outcomes such as clerkship assessment, performance on our institutional standard undergraduate standardized patient assessments, and the medical licensing exam Step 2-clinical skills.  We also plan to adapt the cases slightly and use the OCAT to examine OB-GYN and Family Practice resident communication performance. To our knowledge, ours is the first dedicated difficult OB communication module and focused assessment measure for undergraduate medical students. OB communication modules may offer benefit not only for overall patient-physician interaction but also for improved medical student experiences in their OB/GYN clerkships by enhancing their OB communication skills and sensitivity before they arrive on the wards.

## Conclusions

In four novel difficult OB communication scenarios, the OCAT demonstrates acceptable to excellent IC, but poor IRR due to systematic differences in evaluator rigor. Our ongoing studies focus on optimizing SP training to improve the IRR when using OCAT.

Communication training is required for medical students, and may improve their capacity to provide excellent patient-centered care. The skills to navigate sensitive issues are not obtained through practice of straightforward clinical cases. Obstetrics affords rich material for sensitive and difficult communication scenarios and is generally under-represented in undergraduate medical communication education. Improving communication in the most difficult obstetric scenarios may have a beneficial effect across all specialties and better prepare students for high-risk communication, including delivering the diagnosis of chronic illness, imparting news of significant morbidity or death, discussing suspicion of child abuse, and navigating sensitive social contexts. Many specialties in medicine are impacted by difficult communication, warranting rigorous educational research and integration of challenging communication training in medical curricula. Developing a validated OB communication tool is an important and necessary initial step in evaluating focused OB communication training interventions that can be used to enhance the skills and experience of undergraduate medical students in their women’s care clinical clerkships.  

### Acknowledgements

This project was supported by the Colorado Chapter of the March of Dimes and Department of Obstetrics and Gynecology of the University of Colorado. KJH was supported by the University of Colorado Women's Reproductive Health Research award (K12HD001271). Statistical analysis was supported in part by NIH/NCATS Colorado CTSI Grant Number UL1TR000154. Supporting organizations in no way influenced the scientific process of this paper.

We acknowledge the Center for Advancing Professional Excellence (CAPE) and staff, and the March of Dimes and their families for their interest and support. We thank Drs. Nanette Santoro, Sonya Erickson, and Torri Metz, and Ms. Jennifer LaBudde for helpful discussions of the project and manuscript suggestions.  We also thank Dr. Kristina Tocce for the opportunity to perform the initial pilot run.

### Conflict of Interest

The authors declare that they have no conflict of interest.

## Supplementary material

Supplementary file 1 Standardized Patient Case Summaries

Supplementary file 2 OB Communication Assessment Tool (OCAT): Common items

Supplementary file 3 Communication assessment tool: Case-specific items

## References

[1] Woolley FR, Kane RL, Hughes CC, Wright DD (1978). The effects of doctor--patient communication on satisfaction and outcome of care.. Soc Sci Med.

[2] Venetis MK, Robinson JD, Turkiewicz KL, Allen M (2009). An evidence base for patient-centered cancer care: a meta-analysis of studies of observed communication between cancer specialists and their patients.. Patient Educ Couns.

[3] Street RL, Makoul G, Arora NK, Epstein RM (2009). How does communication heal? Pathways linking clinician-patient communication to health outcomes.. Patient Educ Couns.

[4] Makoul G (2001). Essential elements of communication in medical encounters: the Kalamazoo consensus statement.. Acad Med.

[5] Medical School Objectives Project. Report III, Con-temporary issues in medicine: communication in medicine. Washington, DC: Association of American Medical Colleges [AAMC]; 1999.

[6] Kurtz SM, Silverman JD (1996). The Calgary-Cambridge Referenced Observation Guides: an aid to defining the curriculum and organizing the teaching in communication training programmes.. Med Educ.

[7] Casey PM, Goepfert AR, Espey EL, Hammoud MM, Kaczmarczyk JM, Katz NT, Neutens JJ, Nuthalapaty FS, Peskin E (2009). To the point: reviews in medical education--the Objective Structured Clinical Examination.. Am J Obstet Gynecol.

[8] Huntley CD, Salmon P, Fisher PL, Fletcher I, Young B (2012). LUCAS: a theoretically informed instrument to assess clinical communication in objective structured clinical examinations.. Med Educ.

[9] Cohen DS, Colliver JA, Marcy MS, Fried ED, Swartz MH (1996). Psychometric properties of a standardized-patient checklist and rating-scale form used to assess interpersonal and communication skills.. Acad Med.

[10] Edgcumbe DP, Silverman J, Benson J (2012). An examination of the validity of EPSCALE using factor analysis.. Patient Educ Couns.

[11] Gallagher TJ, Hartung PJ, Gregory SW (2001). Assessment of a measure of relational communication for doctor-patient interactions.. Patient Educ Couns.

[12] Gallagher TJ, Hartung PJ, Gerzina H, Gregory SW, Merolla D (2005). Further analysis of a doctor-patient nonverbal communication instrument.. Patient Educ Couns.

[13] Fossli Jensen B, Gulbrandsen P, Benth JS, Dahl FA, Krupat E, Finset A (2010). Interrater reliability for the Four Habits Coding Scheme as part of a randomized controlled trial.. Patient Educ Couns.

[14] Lang F, McCord R, Harvill L, Anderson DS (2004). Communication assessment using the common ground instrument: psychometric properties.. Fam Med.

[15] Guiton G, Hodgson CS, Delandshere G, Wilkerson L (2004). Communication skills in standardized-patient assessment of final-year medical students: a psychometric study.. Adv Health Sci Educ Theory Pract.

[16] Silverman J, Archer J, Gillard S, Howells R, Benson J (2011). Initial evaluation of EPSCALE, a rating scale that assesses the process of explanation and planning in the medical interview.. Patient Educ Couns.

[17] Wetzel AP (2012). Factor analysis methods and validity evidence: a review of instrument development across the medical education continuum.. Acad Med.

[18] Rosenbaum ME, Ferguson KJ, Lobas JG (2004). Teaching medical students and residents skills for delivering bad news: a review of strategies.. Acad Med.

[19] Makoul G (1998). Medical student and resident perspectives on delivering bad news.. Acad Med.

[20] Kiluk JV, Dessureault S, Quinn G. Teaching medical students how to break bad news with standardized patients. J Cancer Educ. 2012;27(2):277-80. 10.1007/s13187-012-0312-9 PMC4504018 22314793

[21] Kretzschmar RM (1978). Evolution of the Gynecology Teaching Associate: an education specialist.. Am J Obstet Gynecol.

[22] Kitzinger S (2007). Sheila Kitzinger's letter from Europe: the making of an obstetrician.. Birth.

[23] Jabeen D (2013). Use of simulated patients for assessment of communication skills in undergraduate medical education in obstetrics and gynaecology.. J Coll Physicians Surg Pak.

[24] Colletti L, Gruppen L, Barclay M, Stern D (2001). Teaching students to break bad news.. Am J Surg.

[25] Kurtz S, Silverman J, Benson J, Draper J (2003). Marrying content and process in clinical method teaching: enhancing the Calgary-Cambridge guides.. Acad Med.

[26] Rodriguez A, Fisher J, Broadfoot K, Hurt KJ. A challenging obstetric communication experi-ence for undergraduate medical education using standardized patients and student self-reflection. MedEdPORTAL Publications [Inter-net]. 2015 [cited 24 April 2016]; Available from: http://dx.doi.org/10.15766/mep_2374-8265.10121.

[27] Nunnally JC, Bernstein IH. Psychometric theory. 3rd ed. New York: McGraw-Hill; 1994.

[28] Cronbach LJ (1951). Coefficient alpha and the internal structure of tests.. Psychometrika.

[29] Feldt LS, Woodruff DJ, Salih FA (1987). Statistical Inference for Coefficient Alpha.. Applied Psychological Measurement.

[30] Bartko JJ (1966). The intraclass correlation coefficient as a measure of reliability.. Psychol Rep.

[31] Shrout PE, Fleiss JL (1979). Intraclass correlations: uses in assessing rater reliability.. Psychol Bull.

[32] Stuart A (1955). A Test for Homogeneity of the Marginal Distributions in a Two-Way Classification.. Biometrika.

[33] Maxwell AE (1970). Comparing the classification of subjects by two independent judges.. Br J Psychiatry.

[34] R Development Core Team. R: A language and environment for statistical computing [software]. Vienna, Austria: R Foundation for Statistical Computing. 2014 [cited 10 March 2015]; Available from: http://www.R-project.org.

[35] Revelle W. R Package Psych: procedures for psychological, psychometric, and personality research. Version 1.4.5 [software]. 2014 [cited 10 March 2015]; Available from: http://personality-project.org/r/psych.

[36] Gamer M, Lemon J, Singh IFP. R Package irr: various coefficients of interrater reliability and agreement. Version 0.84 [software]. 2012 [cited 15 March 2015]; Available from: http://CRAN.R project.org/package=irr.

[37] Fletcher I, Mazzi M, Nuebling M (2011). When coders are reliable: the application of three measures to assess inter-rater reliability/agreement with doctor-patient communication data coded with the VR-CoDES.. Patient Educ Couns.

[38] Furman GE (2008). The role of standardized patient and trainer training in quality assurance for a high-stakes clinical skills examination.. Kaohsiung J Med Sci.

[39] May W (2008). Training standardized patients for a high-stakes Clinical Performance Examination in the California Consortium for the Assessment of Clinical Competence.. Kaohsiung J Med Sci.

[40] Krupat E, Frankel R, Stein T, Irish J (2006). The Four Habits Coding Scheme: validation of an instrument to assess clinicians' communication behavior.. Patient Educ Couns.

[41] Kevelighan, Duffy, Walker (1998). Innovations in teaching obstetrics and gynaecology - the Theme Afternoon.. Med Educ.

[42] Coonar AS, Dooley M, Daniels M, Taylor RW (1991). The use of role-play in teaching medical students obstetrics and gynaecology.. Med Teach.

[43] Hendrickx K, De Winter B, Tjalma W, Avonts D, Peeraer G, Wyndaele JJ (2009). Learning intimate examinations with simulated patients: the evaluation of medical students' performance.. Med Teach.

[44] Jackson MB, Keen M, Wenrich MD, Schaad DC, Robins L, Goldstein EA (2009). Impact of a pre-clinical clinical skills curriculum on student performance in third-year clerkships.. J Gen Intern Med.

