# Supplementary material for: Psychometric characterization of the obstetric communication assessment tool for medical education: a pilot study
Source: Int J Med Educ. 2016 Jun 11;7:168–79. doi: 10.5116/ijme.5740.4262 (PMC4912696; doi:10.5116/ijme.5740.4262)
Supplement: Supplementary file 1 — Standardized Patient Case Summaries [file ijme-7-168-S1.pdf]

**Appendix 1.**

**Standardized Patient Case Summaries**

| | |
|---|---|
| Case 1<br>Religious Beliefs (RB) | **SUMMARY:** Female patient at 22 weeks gestational age (GA) presenting to Labor and Delivery (L&D) with signs and symptoms of pre-eclampsia with severe features and Hemolysis, Elevated Liver Enzymes, and Low Platelets (HELLP) Syndrome. Discussion of induction of labor (IOL) of a non-viable pregnancy is complicated by the patient's devout religious beliefs and aversion to pregnancy termination.<br><br>**OBJECTIVES:** Deliver difficult news to a patient. Acknowledge and explore patient's religious objection in a non-judgmental manner. |
| Case 2<br>Angry Father (AF) | **SUMMARY:** Female patient at 33 weeks GA presenting to the hospital in active labor, accompanied by her husband, who is angered by feelings of neglect by the hospital staff and in interacting with a medical student.<br><br>**OBJECTIVE:** Acknowledge the partner's feelings in a respectful manner. Manage clinical encounter despite interruption and negative emotional state. |
| Case 3<br>Maternal Smoking (MS) | **SUMMARY:** Female patient at 39 weeks GA presenting to L&D for elective IOL for poorly controlled diabetes. After delays in her induction, the patient threatens to leave if the medical student will not agree to let her smoke.<br><br>**OBJECTIVE:** Manage a difficult patient without acquiescing to inappropriate patient demands. |
| Case 4<br>Intimate Partner Violence (IPV) | **SUMMARY:** Female patient at 31 weeks GA presenting to L&D with complaints of abdominal pain, contractions, and vaginal bleeding following an episode of domestic abuse by her current partner.<br><br>**OBJECTIVE:** Identify presence and character of intimate partner violence. Conduct a meaningful discussion of establishing patient's safety and options available to the patient. |
