# Supplementary material for: Psychometric characterization of the obstetric communication assessment tool for medical education: a pilot study
Source: Int J Med Educ. 2016 Jun 11;7:168–79. doi: 10.5116/ijme.5740.4262 (PMC4912696; doi:10.5116/ijme.5740.4262)
Supplement: Supplementary file 2 — OB Communication Assessment Tool (OCAT): Common items [file ijme-7-168-S2.pdf]

Appendix 2.

OB Communication Assessment Tool (OCAT): Common items

1. Initiating the Session (IS)
2. Greets patient and obtains patient's name or uses name in greeting.
3. Introduces self and role as a medical student.
4. Identifies the patient's problems or the issues that the patient wishes to address with appropriate open-ended question.
5. Demonstrates respect and interest in the initiation of the encounter.

Gathering Information (GI)

1. Encourages patient's story primarily through open-ended questions.
2. Listens attentively, allowing patient to complete statements without interruption and allowing for pauses in conversation.
3. Facilitates patient's responses verbally and non-verbally (may use encouragement, silence, repetition, paraphrasing, and/or interpretation).
4. Elicits the patient's perspective (ideas, beliefs, concerns, OR expectations).
5. Acknowledges patient's verbal and non-verbal cues (body language, speech, facial expression, affect).
6. Elicits patient's biomedical history (obstetric, gynecologic, past medical, surgical and family histories, substance use, medications, and allergies).
7. Elicits patient's background and life context.

Building the Relationship (BR)

1. Demonstrates empathy to communicate understanding and appreciation of patient's feelings or predicament.
2. Accepts patient views and feelings non-judgmentally.
3. Expresses concern and willingness to help; offers partnership.
4. Demonstrates appropriate non-verbal behaviors, including eye contact, facial expression, and vocal cues.
5. If reads or writes notes, does so in a manner that does not interfere with dialog or rapport. If the learner DID NOT use notes or paper during the encounter, select N/A.
6. Communicates in a way that is non-judgmental, supportive, and sympathetic.

Providing Structure (PS)

1. Progresses from one section to another using signposting and/or transitional statements.
2. Attends to timing, keeping on task as appropriate (without rushing).

Sharing Information (SI)

1. Elicits patient understanding of illness or situation.
2. Asks about patient support systems.
3. Provides opportunities and encourages patient to contribute (ask questions, seek clarification or express doubts).
4. Addresses the fetus or fetal problem/prognosis as a separate concern, issue, or perspective.
5. Partners with patient to tailor a suitable plan of action, discussing impact, support, and responsibilities.

Closing the Session (CS)

1. Summarizes visit appropriately and clarifies plan (or next steps).
2. Final check that patient agrees and is comfortable with the plan.

Overall Student Performance (OSP)

1. I trust the student-doctor to act as an advocate for my health and well-being. If selecting "No" please respond in the text box below
 as to the reason for the selection.*
2. As a patient, I would gladly, agree to, or prefer not to interact with the student-learner again (please select one below).If selecting
 an option other than 'gladly agree', please use the text box below to state reason for your selection.

*Denotes question with response of "yes" or "no".
