# Supplementary material for: Psychometric characterization of the obstetric communication assessment tool for medical education: a pilot study
Source: Int J Med Educ. 2016 Jun 11;7:168–79. doi: 10.5116/ijme.5740.4262 (PMC4912696; doi:10.5116/ijme.5740.4262)
Supplement: Supplementary file 3 — Communication assessment tool: Case-specific items [file ijme-7-168-S3.pdf]

Appendix 3.

Communication assessment tool: Case-specific items

Religious Beliefs (RB)

1. Does the learner identify the connection between religion and the patient's preferences regarding her care?* (GI)
2. Explores all options (Specifically, addresses possible actions and alternatives for management of patient's condition. See below for options discussed.) (SI)
3. Was there a discussion of options available to the patient in regards to possible actions and alternatives for management of patient's condition?* (SI)
4. Did the learner and patient discuss the option of no medical action: patient refuses medical care and pursues prayer for relief of condition?* (SI)
5. Did the learner and patient discuss the option of patient consenting to medical care for her condition?* (SI)
6. Did the learner and patient discuss the option of patient delays consent for procedures until she is able to speak with her husband and/or family?* (SI)
7. Did the learner and patient discuss the option of patient delays consent for procedures until she is able to speak with religious figure (hospital's chaplain, priest from her congregation, etc.)?* (SI)
8. Did the learner and patient discuss any other option? If yes, please use the text box below to list additional options discussed* (SI)

Angry Father (AF)

1. Acknowledges the patient's partner and initiates necessary introductions. (IS)
2. Did the student acknowledge the partner's feelings and/or address this as a separate issue?* (BR)
3. Did the student redirect the partner as appropriate?* (BR)
4. Student addresses the partner's concerns and comments in a meaningful way (BR)
5. Managed flow of interview with patient despite interruption/interference from partner (PS)

Maternal Smoking (MS)

1. Does the learner identify patient's current smoking habits?* (GI)
2. Explores all options (Specifically, addresses possible actions and alternatives to smoking in the hospital. See below to select options discussed.) (SI)
3. Was there a discussion of options available to the patient in response to requests to smoke?* (SI)
4. Did the learner agree to the option of patient leaving the hospital room to smoke?* (SI)
5. Did the learner and patient discuss the option of a smoking alternative (nicotine patch, gum, lozenge)?* (SI)
6. Did the learner and patient discuss the option of being denied her request/demand to smoke without the offer of a smoking alternative?* (SI)
7. Did the learner and patient discuss any other option(s)? If "Yes" please use the text box below to list additional options discussed.* (SI)
8. Assess patient's willingness to quit smoking (may include asking the patient if she is willing to quit smoking, offering resources on smoking cessation, etc.) (SI)

Intimate Partner Violence (IPV)

1. Identifies patient is a victim of Intimate Partner Violence.* (GI)
2. Asks questions re: patient safety, (Ex: Do you feel safe at home and in your relationships? OR Do you have a safe place to stay?)* (GI)
3. Explores all options (Specifically, addresses possible actions and alternatives for IPV. See below for options discussed.) (SI)
4. Was there a discussion of options available to the patient?* (SI)
5. Did the learner and patient discuss the option of no action or patient remaining with abusive partner?* (SI)
6. Did the learner and patient discuss the option of patient permanently leaving the abusive partner immediately or in the future?* (SI)
7. Did the learner and patient discuss the option of patient contacting law enforcement?* (SI)
8. Did the learner and patient discuss the option of patient accessing resources, such as counseling (individual or family), battered women's shelter, or support group?* (SI)
9. Did the learner and patient discuss any other option(s)? If "Yes" please use the text box below to list additional options discussed.* (SI)
10. Establishes patient's safety upon reintroduction to the home environment. (Ex: Who will you call if this happens again? OR Is there a place you can take yourself and your children where you will be safe?) (SI)

*Denotes question with response of "yes" or "no".
Parentheses denote subscale assignment for each item: Gathering Information (GI), Sharing Information (SI), Building the Relationship (BR), and Providing Structure (PS).
